# Vaptans: A Narrative Review of Pharmacokinetics, Pharmacogenetics, and Clinical Applications

**DOI:** 10.3390/cells15151388

**Published:** 2026-07-31

**Authors:** Martyna Lica-Miler, Karol Miler, Dorota Kostrzewa-Nowak, Andrzej Ciechanowicz, Andrzej Brodkiewicz, Jacek Różański, Joanna Stępniewska

**Affiliations:** 1 Department of Nephrology, Transplantology and Internal Medicine, Pomeranian Medical University, Powstańców Wielkopolskich 72, 70-111 Szczecin, Poland; martyna.lica00@gmail.com (M.L.-M.); joanna.stepniewska@pum.edu.pl (J.S.); 2 Department of Clinical and Molecular Biochemistry, Pomeranian Medical University, Powstańców Wielkopolskich 72, 70-111 Szczecin, Poland; kmiler01@gmail.com (K.M.); dorota.kostrzewa.nowak@pum.edu.pl (D.K.-N.); andrzej.ciechanowicz@pum.edu.pl (A.C.); 3 Clinic of Pediatrics, Pediatric Nephrology, Dialysis and Treatment of Acute Poisoning, PUM Clinic in Szczecin at the “Zdroje” Hospital, ul. Maczna 4, 70-780 Szczecin, Poland; andrzej.brodkiewicz@pum.edu.pl

**Keywords:** vaptans, pharmacogenetics, tolvaptan, pharmacokinetics, ADPKD

## Abstract

Vaptans are non-peptide arginine vasopressin (AVP) receptor antagonists essential for modulating water homeostasis. This review evaluates their chemical evolution from peptide analogs to orally bioavailable molecules and summarizes the pharmacokinetic profiles of key agents, including tolvaptan, conivaptan, and mozavaptan. Significant emphasis is placed on the clinical role of tolvaptan as a disease-modifying therapy in autosomal dominant polycystic kidney disease (ADPKD) and its efficacy in syndrome of inappropriate antidiuretic hormone secretion (SIADH). Furthermore, the impact of pharmacogenetic factors, especially *CYP3A5* and *SVEP1* polymorphisms, is explored as a potential source of inter-individual variability in treatment response. By integrating structural, metabolic, and genetic data, this article highlights the potential for personalized aquaretic therapy and identifies critical safety and monitoring protocols necessary for optimizing clinical outcomes.

## 1. Introduction

**Figure 1:**
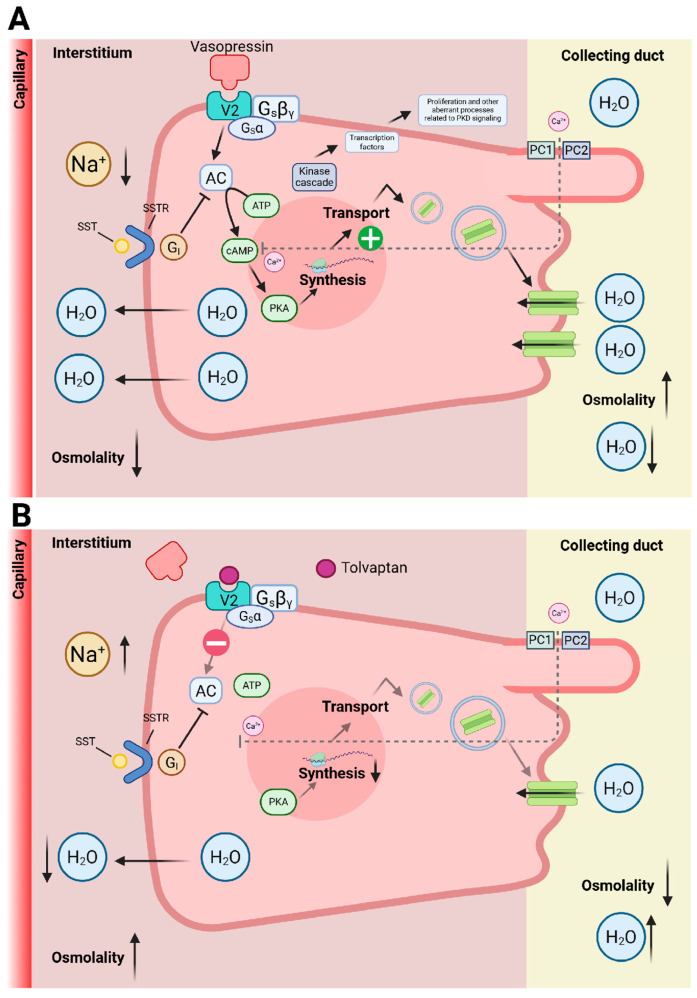
Pathomechanism of ADPKD and the Mechanism of Action of Tolvaptan in Renal Collecting Duct Cells. (**A**) Simplified pathophysiology of ADPKD. Vasopressin binds to the vasopressin V2 receptor, activating heterotrimeric G proteins that stimulate adenylyl cyclase (AC). AC catalyzes the conversion of ATP to cyclic AMP (cAMP), leading to the downstream activation of protein kinase A (PKA). This signaling pathway enhances water reabsorption and drives a phosphorylation-dependent kinase cascade that activates transcription factors responsible for aberrant cell proliferation and impaired tubulogenesis. (**B**) Mechanism of action of Tolvaptan. Tolvaptan competitively inhibits the binding of vasopressin to the V2 receptor, which suppresses the processes described above and slows down cystogenesis (or: aberrant tubulogenesis). Figure adapted and simplified from multiple sources [11,12,13,14]. Created with BioRender. Sławiński, M. (2026) https://www.biorender.com/ (accessed on 27 July 2026).

Vasopressin, also known as antidiuretic hormone (ADH) [1], is a fundamental regulator of the body’s water balance. It is synthesized in the hypothalamus and subsequently released from the posterior pituitary gland in response to increased plasma osmolality. ADH binds to V2 receptors on the basolateral membrane of the renal collecting duct, initiating a signaling cascade mediated by adenylate cyclase and cyclic adenosine monophosphate (cAMP). This process triggers the translocation of vesicles containing aquaporin-2 (AQP2) to the apical membrane of the cell. These aquaporins form channels through which water is reabsorbed from the urine back into the bloodstream, resulting in increased urine concentration and expanded plasma volume [2] (Figure 1A).

Vaptans represent a class of arginine vasopressin (AVP) receptor antagonists. They exert their effect through the competitive displacement of vasopressin from its binding sites. To date, clinical trials have evaluated the efficacy of several molecules within this group, including mozavaptan, conivaptan, lixivaptan, satavaptan, and tolvaptan [3,4,5] (Figure 1B). However, the regulatory status of these agents currently varies by region. Tolvaptan and conivaptan have received U.S. Food and Drug Administration (FDA) approval for the treatment of clinically significant hypervolemic and euvolemic hyponatremia. In contrast, within the European Union, only tolvaptan has been authorized by the European Medicines Agency (EMA) for the management of the syndrome of inappropriate antidiuretic hormone secretion (SIADH) and autosomal dominant polycystic kidney disease (ADPKD) [6,7]. Due to safety concerns, insufficient clinical benefit, and a lack of impact on mortality in the treatment of heart failure and liver cirrhosis, the use of satavaptan and lixivaptan has been restricted to clinical trials [8,9]. Since the inception of research into these agents, they have been associated with high expectations regarding their therapeutic potential [10]. Given its broad regulatory profile, tolvaptan remains the most extensively studied representative of this class. It is the primary focus of the present paper and continues to be one of the most promising agents for further investigation.

## 2. Literature Search Strategy

This narrative review was based on a non-systematic literature search performed using the PubMed/MEDLINE and Google Scholar databases. Searches were conducted using combinations of keywords related to the scope of the review, including “tolvaptan”, “vaptans”, “vasopressin receptor antagonists”, “autosomal dominant polycystic kidney disease”, “ADPKD”, “hyponatremia”, “heart failure”, “SIADH”, “pediatrics” and related terms. Priority was given to recent publications whenever possible; however, seminal and landmark studies were also included to provide historical context and illustrate the evolution of vaptan therapy. Additional relevant articles were identified through manual screening of the reference lists of selected publications.

## 3. Characteristic of Vaptans

### 3.1. Tolvaptan

Tolvaptan (OPC-41061) [15] is an arginine vasopressin receptor type 2 (V2R) antagonist. It induces increased excretion of solute-free water (aquaresis) without disrupting electrolyte balance [16]. Following oral administration, tolvaptan selectively antagonizes vasopressin receptors within the renal collecting ducts [10,17,18]. The pharmacokinetic profiles and the corresponding pharmacodynamic potency of tolvaptan following a single oral dose were previously elucidated by Shoaf et al. [19]. The diuretic effect during the initial 12 h post-administration did not vary significantly across doses. However, within the 0 to 72 h interval, it increased linearly with single doses ranging from 60 mg to 480 mg, attributable to prolonged drug concentration in the systemic circulation. Notably, a relative concentration plateau was achieved only at a single dose of 300 mg. Plasma concentrations of tolvaptan elicited a maximal response even at the lowest evaluated dose 60 mg, which facilitated the determination of the maximum routinely administered dose.

Tolvaptan reaches peak plasma concentration approximately 2–4 h following oral administration [20], with an absolute bioavailability of approximately 56%. It undergoes extensive hepatic metabolism mediated by the CYP3A4 cytochrome isoenzymes, and its metabolites are primarily excreted via the feces [21]. The drug is degraded into several metabolites [22], the two most significant of which are identified as the oxobutyric acid metabolite (DM-4103) and the hydroxybutyric acid metabolite (DM-4107) [23]. The predominant metabolite, DM-4103, exhibits no affinity for V2 receptors; however, it possesses hepatotoxic potential [23,24]. During treatment, the concomitant administration of moderate-to-potent CYP3A inhibitors and inducers should be avoided [21,25]. Furthermore, caution is warranted when co-administering P-glycoprotein (P-gp) substrates [26], due to the risk of elevated drug concentrations resulting in increased urinary output, or conversely, diminished concentrations leading to reduced therapeutic efficacy. Recent molecular studies have demonstrated that the pharmacological activity of V2 receptor antagonists depends not only on their equilibrium binding affinity but also on ligand–receptor binding kinetics [27]. In a kinetic study on the human V2 receptor, Liu et al. demonstrated that individual vaptans differ considerably in their residence time, showing that lixivaptan exhibits a relatively long receptor residence time compared to the rapid dissociation observed for mozavaptan. At the structural level, Cryo-EM studies by Zhang et al. [28] revealed the atomic details of antagonist binding within the V2 receptor pocket. Specifically, tolvaptan occupies a deep orthosteric binding pocket, whereas conivaptan adopts a shallower binding orientation. Furthermore, structural analyses demonstrated that antagonist binding induces a specific conformational shift in transmembrane helix 7 (TM7), establishing a TM7-dependent mechanism of V2 receptor inactivation. Together, these kinetic and structural insights highlight how differences in binding depth and receptor–ligand kinetics contribute to the distinct pharmacological profiles of individual vaptans.

### 3.2. Conivaptan

In contrast to selective V2R antagonists (such as tolvaptan or lixivaptan), conivaptan (YM087) [29] acts as a dual antagonist of both V1a and V2 receptors. Conivaptan is primarily administered intravenously, a formulation marketed under the trade name Vaprisol. Following both oral and intravenous administration, the peak diuretic effect is attained between two and four hours post-dose [30], with the duration of the aquaretic effect lasting approximately 12 h after a single dose. Conivaptan exhibits non-linear pharmacokinetics at daily doses of 40 mg/d and 80 mg/d, due to mechanism-based self-inhibition of its CYP3A4-mediated metabolism. Although initially evaluated in both formulations, the oral route was discontinued after pharmacokinetic studies revealed that this potent inhibition led to marked drug accumulation and a high risk of severe drug–drug interactions [31,32]. Consequently, conivaptan is contraindicated in patients receiving other strong CYP3A4 inhibitors. The aforementioned oral pharmacokinetic data reflect findings from earlier clinical trials preceding the discontinuation of this formulation. The majority of the drug is excreted via the feces (83%). The impact of hepatic or renal impairment on the pharmacokinetics of conivaptan has not been sufficiently evaluated, primarily due to its short-term administration and a paucity of clinical studies.

### 3.3. Mozavaptan

A significant milestone in the pursuit of effective methods for modulating body water homeostasis was the development of the first class of non-peptide vasopressin receptor antagonists. Among these, mozavaptan (Physuline, OPC-31260), a selective V2 receptor antagonist, exhibits the highest structural homology to tolvaptan. Although its pharmacological profile was elucidated as early as 1992 [33], making it the first characterized non-peptide V2R antagonist, it was not officially approved for clinical use in Japan until 2006. Clinical trials have demonstrated that this agent increases urinary output in a dose-dependent manner, particularly within the first 4 h post-administration, eliciting an aquaretic effect with minimal impact on electrolyte excretion [34]. The peak diuresis and the minimum urinary osmolality were observed between 60 and 90 min following administration. Due to its close structural similarity to tolvaptan, mozavaptan has also served as a key model in cellular immunological research; specifically, recent findings demonstrated that tolvaptan-responsive T-cell clones can be cross-activated by mozavaptan, revealing a shared structural antigenicity and T-cell-mediated immune mechanism relevance [35].

### 3.4. Satavaptan and Lixivaptan

Satavaptan (SR-121463) is a selective V2 receptor antagonist with a terminal half-life ranging from 14 to 17 h [36,37]. Peak plasma concentrations are typically observed 3 h post-administration; these levels increase in a dose- and time-dependent manner, reaching a steady state by the fifth day of treatment. Notably, in patients receiving concomitant carbamazepine, which is a known CYP3A inducer, plasma concentrations of satavaptan were markedly lower.

Lixivaptan (VPA-985) represents another selective V2 receptor antagonist. Preclinical animal studies have demonstrated that this agent is highly effective following oral administration, exhibiting significantly greater pharmacological potency than its prototype predecessor, OPC-31260 [38]. Although previous clinical studies in patients with euvolemic hyponatremia and cirrhosis suggested that lixivaptan exerts an aquaretic effect, direct evidence demonstrating its action on vasopressin-induced aquaporin-2 (AQP2) trafficking in collecting duct principal cells was lacking. This gap was addressed by an in vitro study showing that lixivaptan inhibits vasopressin-induced cAMP signaling, prevents AQP2 translocation to the apical membrane, and reduces osmotic water permeability without affecting AQP2 expression, thereby providing direct mechanistic evidence for its aquaretic activity [9]. A summary of all vaptans is provided in Table 1.

## 4. The Role of Vaptans in Disease Management

### 4.1. Autosomal Dominant Polycystic Kidney Disease

**Figure 2:**
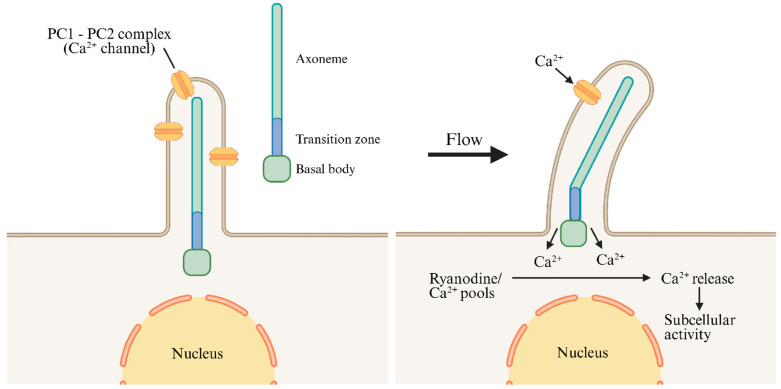
Schematic representation of renal tubular epithelial cell function. A primary cilium with a sensory function is located on the apical surface. Embedded within the ciliary membrane is the Polycystin-1–Polycystin-2 (PC1–PC2) complex, which functions as a mechanosensitive calcium channel. Left panel: In the absence of fluid flow within the renal tubule, the primary cilium remains upright, and the PC1–PC2 channel remains closed. Basal transcriptional activity maintains the expression of genes that suppress excessive cellular proliferation. Right panel: The flow of primary urine through the tubule generates shear stress, causing deflection (bending) of the primary cilium and opening of the PC1–PC2 channel. This enables the influx of Ca^2+^ ions from the extracellular space into the cell, initiating the release of substantially larger amounts of Ca^2+^ from intracellular stores, particularly from ryanodine receptor-sensitive calcium pools. The resulting amplified calcium signal activates downstream intracellular signaling pathways that are essential for normal cellular and renal function.

Autosomal dominant polycystic kidney disease (ADPKD) is the most common hereditary renal disease. It is characterized by an autosomal dominant inheritance pattern [39,40], although de novo mutations may also occur [41]. The genetic etiology primarily involves mutations in the *PKD1* (75–85%) and *PKD2* (15%) genes [42]. The hallmark of ADPKD is the formation of numerous renal cysts, which lead to progressive renal enlargement and structural parenchymal damage. A more detailed description of cystogenesis in ADPKD is provided under Figure 2. Hypertension frequently manifests as the initial clinical symptom. As the disease progresses, it leads to advanced chronic kidney disease (CKD), ultimately necessitating renal replacement therapy (RRT). Extrarenal manifestations include, among others, hepatic cysts and intracranial aneurysms. The diagnostic criteria for ADPKD are based on imaging and genetic testing, integrated with the patient’s age and family history. Ultrasonography remains the primary clinical tool, utilizing age-dependent cyst count criteria to establish a diagnosis [43]. While the cornerstone of ADPKD management involves blood pressure control, symptomatic treatment, and interdisciplinary care [44], tolvaptan is currently the only pharmacological agent with proven efficacy in slowing disease progression.

In autosomal dominant polycystic kidney disease, arginine vasopressin exerts a pathological effect through the activation of V2 receptors, which stimulates cAMP-dependent ERK (Extracellular Signal-Regulated Kinase) activity. This process accelerates renal cyst growth and epithelial cell proliferation. As an AVP receptor blocker, tolvaptan effectively slows this phenomenon [14]. Tolvaptan is unique among vaptans as the first and only disease-modifying drug approved for ADPKD. Its international regulatory status was established through pivotal Phase 3 and Phase 3b clinical trials. The landmark TEMPO 3:4 trial (Phase 3) [45] published in 2012, demonstrated that tolvaptan slows the increase in total kidney volume and reduces ADPKD-related complications (hypertension, pain, hematuria, kidney stones) compared to placebo. Based primarily on these findings, the European Medicines Agency (EMA) approved tolvaptan for ADPKD in 2015. The most significant effect was observed during the first year of treatment, although the benefits remained evident throughout the second and third years of observation. The trial included patients in the early stages of ADPKD who had a TKV ≥ 750 mL and eGFR ≥ 60 mL/min/1.73 m^2^.

The next step involved evaluating the drug’s efficacy in patients at later stages of the disease, which was addressed in the multinational Phase 3 REPRISE trial [46]. This study demonstrated that tolvaptan slows the loss of renal function in patients with advanced disease, specifically those aged 18 to 55 with an estimated GFR (eGFR) between 25 and 65 mL/min/1.73 m^2^, or those aged 56 to 65 with an eGFR between 25 and 44 mL/min/1.73 m^2^. The observed effects were similar to those seen in the group with early stage ADPKD. The positive outcomes of the REPRISE trial provided the critical evidence required for approval by the U.S. Food and Drug Administration (FDA) in 2018. To assess the long term stability of these effects and the risk of relapse, a cohort of patients from the TEMPO 3:4 trial was followed in the TEMPO 4:4 program (an open-label Phase 3b extension study) [47], which monitored treatment efficacy for an additional two years. The study had certain limitations, including treatment interruptions and the loss of randomization. The results did not show a sustained benefit regarding the difference in total kidney volume (TKV) achieved in the previous study. However, the trial demonstrated a durable beneficial effect on eGFR, slowing its decline as effectively as in the TEMPO 3:4 trial. This confirmed the disease-modifying action of the drug, highlighting the advantages of early initiation and long-term treatment.

To determine the optimal dosing, a gradual dose titration strategy was implemented from the initial clinical trials. Three dual-dose regimens, administered in the morning and evening, were proposed: 45/15 mg, 60/30 mg, and 90/30 mg, depending on patient tolerance. Current KDIGO guidelines recommendations about initiating tolvaptan therapy in ADPKD patients are summarized in Table 2.

In ADPKD, mutations in the PKD1 (encoding PC1) or PKD2 (encoding PC2) genes result in the absence or dysfunction of the PC1–PC2 complex. Consequently, deflection of the primary cilium by fluid flow fails to open the channel, rendering the cell unable to sense mechanical stimuli. The absence of Ca^2+^ influx disrupts the entire signaling cascade. The loss of physiological calcium signaling is interpreted by the cell as a proliferative stimulus and, together with altered ion and water transport, promotes tubular wall outpouching, formation of a closed cyst, and the progressive development of cysts characteristic of ADPKD. Figure adapted and simplified from multiple sources [48,49]. Created in BioRender, 2026. Available online: https://BioRender.com/sd56cdz (accessed on 27 July 2026).

Treatment begins with the lowest recommended dose of 45/15 mg, with a target dose of 90/30 mg or the maximum tolerated dose. A study by Akihisa et al. [50] confirmed that increasing the tolvaptan dose, when adjusted for body weight, positively correlates with eGFR preservation. Conversely, Roca Oporto et al. [51] proposed urinary osmolality as a biomarker to facilitate dose selection. It was noted that patients receiving tolvaptan maintained urinary osmolality below 200 mOsm/kg over a 3 year observation period, even at minimum doses. This represented a significant departure from major trials such as TEMPO 3:4 and REPRISE [45,46], which aimed for the maximum tolerated doses. Evidence suggests that utilizing urinary osmolality as a guide resulted in fewer adverse events and a lower rate of treatment discontinuation due to side effects. These findings highlight the need to consider alternative methods for establishing a safe dosage for patients with rapidly progressing ADPKD [52]. The determination of optimal dosing remains a subject of ongoing debate. A systematic review and meta-analysis of eight studies conducted by Li et al. [53] found no significant differences between various tolvaptan dosages. However, the authors cautioned that these results should be considered preliminary rather than definitive due to insufficient data.

ADPKD is a multisystem disorder that is not confined solely to the kidneys. It is characterized by the frequent co-occurrence of hepatic cysts [54] and intracranial aneurysms [55]. The impact of tolvaptan on hepatic cysts is not clear. A study by Mancinelli et al. [56] demonstrated that, contrary to previous assumptions, vasopressin V2 receptors are expressed in hepatic cysts, and tolvaptan slows the proliferation of these cysts in both murine models and human hepatic cyst models in vitro. Isolated case reports have described a reduction in total liver volume (TLV) during tolvaptan treatment for ADPKD [57]. However, the current lack of data and a lack of dedicated clinical trials preclude the establishment of indications for tolvaptan use solely for the purpose of reducing hepatic cyst burden. Regarding intracranial aneurysms, research has not demonstrated a direct effect of tolvaptan on reducing the incidence of aneurysms or subarachnoid hemorrhage (SAH). Nevertheless, the drug may indirectly mitigate the risk of hemorrhage by slowing the progression of renal failure and slowing cyst proliferation, thereby preventing secondary hypertension. In a 2025 study by Şahin AZ et al. [58], the impact of tolvaptan on systemic inflammation in ADPKD patients was evaluated. Levels of C-reactive protein (CRP), the platelet-to-lymphocyte ratio (PLR), serum uric acid, the systemic inflammatory index (SII), and proteinuria significantly decreased in the tolvaptan group, while these parameters increased in the control group. Although these are isolated findings that require further research, the results are highly promising for the broader management of ADPKD.

### 4.2. Heart Failure

Tolvaptan has also found application in the treatment of heart failure. Diuretic therapy remains one of the fundamental pillars of heart failure (HF) management [59]. Despite the diuretic effect of tolvaptan, it is not routinely used in HF treatment. Tolvaptan offers an advantage over conventional diuretics because it reduces the amount of free water in the body without disrupting electrolyte balance or exerting a dysregulatory effect on the kidneys [16]. Hyponatremia, which frequently co-occurs with HF [60], is correlated with higher readmission rates [61] and prolonged hospital stays. As a selective vasopressin V2 receptor antagonist, tolvaptan increases serum sodium levels, showing additional efficacy in treating both euvolemic and hypervolemic hyponatremia, including in patients with heart failure [62,63,64]; this gives it an advantage over standard diuretics in such cases. However, certain limitations must be considered, such as the need for precise monitoring of the patient’s hydration status during treatment. A significant turning point in the clinical evaluation of tolvaptan was the multicenter EVEREST trial [65]. Although it confirmed the drug’s short-term efficacy in relieving symptoms such as dyspnea and edema, it did not affect primary endpoints, such as mortality. For this reason, tolvaptan and other vaptans are not considered primary pillars in the therapy of heart failure.

Isolated studies have described the impact of tolvaptan on the clinical course of heart failure in cardiac surgery patients undergoing cardiopulmonary bypass (CPB). In these cases, its administration was associated with a rapid reduction in postoperative fluid overload [66] and improved efficacy in postoperative volume management [67,68]. Similarly, in patients undergoing surgery for Stanford type A aortic dissection, the drug’s efficacy was comparable to that of routinely used standard diuretics [69]. Furthermore, a decreased incidence of atrial fibrillation (AF) was noted in patients treated with tolvaptan compared to those receiving conventional diuretics. Similar effects were reported by Nakamura et al. [70], who demonstrated that tolvaptan may significantly reduce the occurrence of postoperative atrial fibrillation by inhibiting the renin–angiotensin–aldosterone system (RAAS) and the sympathetic nervous system compared to control groups. A report by Wu et al. [71] using a mouse model showed that high doses of tolvaptan (10 mg/kg/day) limited the development of abdominal aortic aneurysms (AAA). In an in vitro model using human aortic smooth muscle cells (HASMCs), tolvaptan exerted its effect by inhibiting matrix metalloproteinase (MMP) expression and the apoptosis of stimulated HASMCs. These studies are summarized in Table 3.

The use of other vaptans in the context of heart failure is highly limited. A multicenter study by Ghali et al. [72] evaluated the efficacy of lixivaptan in outpatients with HF. A subset of patients was administered 100 mg of the drug daily and monitored for 8 weeks, with results compared to a placebo group. Lixivaptan was found to reduce dyspnea and edema and was generally well-tolerated. However, despite the relief of short-term symptoms, it was demonstrated that the drug does not provide significant clinical benefits or reduce mortality. Consequently, the development of lixivaptan as a commercially available treatment was discontinued [73].

When interpreting these findings, it is crucial to distinguish between both the strengths of the evidence and the distinct clinical populations studied. The landmark EVEREST trial evaluated patients with chronic, classical heart failure presenting with acute decompensation. In this population, where heart failure is a progressive, long-term disease, tolvaptan showed only short-term symptomatic relief without affecting long-term survival or rehospitalization rates. While these small-scale studies suggest small but significant benefits of tolvaptan in accelerating postoperative fluid removal and reducing the incidence of postoperative arrhythmias, they are inherently limited by observational designs and small sample sizes. Therefore, while tolvaptan shows promise as a specialized tool for acute post-surgical volume management, high-level evidence from the EVEREST trial confirms it does not serve as a primary disease-modifying therapy for classical, chronic heart failure.

### 4.3. Hyponatremia

The vasopressin receptor antagonists conivaptan and tolvaptan are approved by the U.S. Food and Drug Administration (FDA) for the treatment of euvolemic and hypervolemic hyponatremia. A 2018 study by Rajan et al. [74] compared the efficacy of sodium correction in 40 postoperative patients, who were randomly administered either 20 mg of intravenous conivaptan or 15 mg of oral tolvaptan. The results demonstrated that both drugs are effective in treating hyponatremia. However, conivaptan achieved a significantly more rapid correction, with statistical superiority in serum sodium levels observed at both 12 and 24 h post-administration (*p* = 0.041 and *p* = 0.004, respectively) compared to the tolvaptan group, before reaching comparable levels of values in both groups at 48 h.

The initial reports indicating the efficacy of tolvaptan were the SALT-1 and SALT-2 trials in 2006 [64]. These studies demonstrated that the drug effectively increased sodium concentrations by days 4 and 30 of treatment in patients with euvolemic and hypervolemic hyponatremia associated with chronic heart failure, SIADH, or liver cirrhosis. A significant improvement was observed in the tolvaptan group (225 patients) compared to the placebo group (223 patients). A specific example of hyponatremia is the Syndrome of Inappropriate Antidiuretic Hormone Secretion (SIADH). While hypertonic saline (3% NaCl) is reserved for the urgent treatment of acute, severely symptomatic hyponatremia to rapidly elevate serum sodium, fluid restriction—sometimes complemented by physiological saline (0.9% NaCl) in mild-to-moderate asymptomatic cases—serves as the standard first-line intervention [75]. However, nearly half of SIADH patients do not respond adequately to fluid restriction due to poor adherence or impaired renal free-water clearance. In such non-responders, second-line pharmacological strategies are required, including oral urea or targeted V2 receptor antagonists such as tolvaptan, which directly antagonizes AVP actions [76]. It possesses a 1.8 times higher affinity for V2 receptors than vasopressin itself, which results in high diuretic efficacy and water loss. However, a key limitation is the risk of overly rapid correction of natremia, which can lead to osmotic demyelination syndrome (ODS). Therefore, regular monitoring of serum sodium levels and potential dose reduction of tolvaptan are mandatory during treatment [77,78]. Research into the optimal application of tolvaptan is ongoing. However, the benefits appear to outweigh the risks, and this strategy is increasingly used in severe cases of SIADH, including in pediatric patients [79].

Conivaptan (Vaprisol) was approved by the FDA for the treatment of euvolemic hyponatremia in 2005, and its indications were expanded to include hypervolemic hyponatremia in 2007 [80]. Unlike tolvaptan, it has not been approved by the European Medicines Agency (EMA). A 2006 study by Ghali et al. [31] evaluated the safety and efficacy of oral conivaptan in both euvolemic and hypervolemic hyponatremia. In a group of 74 patients, conivaptan was administered at doses of 80 mg/d or 40 mg/d, or as a placebo. In both conivaptan groups, there was a statistically significant increase in serum sodium levels and a faster normalization of natremic parameters compared to the placebo group. In 2007, Zeltser et al. [81] investigated the effects of intravenous conivaptan administered at 40 mg/d in patients with hyponatremia. Their randomized controlled trial demonstrated a significant increase in serum sodium levels, with a mean change from baseline of, depending on the dose, 6.3 mEq/L and 9.4 mEq/L by the end of the 4-day infusion period (*p* < 0.0001), confirming rapid efficacy and good treatment tolerability over a four day observation period. Despite the drug’s efficacy, emphasis is placed on exercising extreme caution during sodium correction, particularly at higher doses of conivaptan (80 mg/d). Clinical studies have documented cases of overly rapid correction, which necessitated the discontinuation of therapy due to the risk of central pontine myelinolysis [82].

Emerging reports indicate the potential for expanding the clinical applications of conivaptan. A pilot study conducted by Corry et al. [83] involving seven patients with cerebral edema secondary to intracerebral hemorrhage confirmed the efficacy and safety of conivaptan over a three month observation period. This study represents a further step toward systematizing knowledge and establishing treatment protocols for managing cerebral edema with this agent. It complements existing evidence based on case reports [84,85] that describe the ability of conivaptan to reduce intracranial pressure (ICP).

The efficacy of oral lixivaptan in the treatment of hyponatremia was evaluated in two pivotal Phase III clinical trials. The HARMONY trial [86], conducted in 2012, assessed the drug’s efficacy in outpatients with euvolemic hyponatremia. The second study, the LIBRA trial [87], focused on hospitalized patients with the same condition. Both were multicenter, randomized, double-blind, placebo-controlled clinical trials. In both cases, positive therapeutic outcomes were achieved lixivaptan significantly increased serum sodium concentrations compared to placebo, while demonstrating a favorable safety profile. This effect was evident from the initial phase of therapy and was sustained throughout the observation period. Despite these positive results, the drug registration process was halted in 2012 due to concerns regarding hepatotoxicity and an unfavorable risk-benefit ratio [88], as well as insufficient clinical advantages compared to conventional diuretics.

Isolated reports evaluate the use of mozavaptan in the Japanese population for SIADH associated with malignant tumors [89]. In one study, a dose of 30 mg/d was administered to 14 patients for up to seven days. The results showed that mozavaptan significantly increased serum sodium levels in the subjects. However, it must be noted that this was a small-scale study, and there is a lack of sufficient data regarding the drug’s efficacy in other populations. Large-scale clinical trials were not implemented, primarily due to the widespread popularity and established role of tolvaptan. Due to the limited number of publications on mozavaptan, comprehensive knowledge regarding its adverse effects remains restricted. During clinical trials, the most frequently reported side effect by patients was dry mouth [34].

Similarly to other V2 receptor antagonists, satavaptan has been the subject of clinical trials focused on the treatment of hyponatremia. A 2011 study by Aronson et al. [90] demonstrated that satavaptan, at doses of both 25 mg and 50 mg, was significantly more effective than placebo in treating hyponatremia caused by congestive heart failure. However, the higher dose was associated with an increased risk of rapid sodium correction among the subjects. The long term effects of satavaptan treatment for hyponatremia caused by SIADH were also evaluated [36] in a study lasting 12 months. The drug effectively increased serum sodium levels and maintained them within the normal range throughout the treatment period. Satavaptan was generally well tolerated, with the most common adverse effects being increased thirst, dry mouth, and frequent urination (polyuria). Nevertheless, the relatively high risk of side effects means that clinical research into the application of vaptans for these conditions is currently less common [8].

### 4.4. Liver Cirrhosis

Tolvaptan has also found application as an adjunct therapy in the treatment of ascites, most commonly caused by liver cirrhosis [91]. In this context, the drug is not the first-line treatment; it is typically preceded by high-dose therapy with other diuretics, primarily furosemide. However, current research suggests that prior use of high-dose furosemide without clinical improvement may negatively impact subsequent tolvaptan treatment [92]. Conversely, earlier initiation of tolvaptan therapy is associated with greater efficacy in reducing ascites [93].

Despite these findings, some experts consider tolvaptan treatment to be unreliable due to its higher cost compared to conventional diuretics and insufficiently superior clinical outcomes. A 2012 meta-analysis [8] indicates that tolvaptan exerts only a minor beneficial effect on hyponatremia and ascites and does not affect the mortality of the study group. Furthermore, attention has been drawn to a higher incidence of bleeding events in patients treated with vaptans. This is supported by preclinical data, which indicate that tolvaptan reduces the concentration of vitamin K-dependent clotting factors and inhibits platelet aggregation [94].

Some studies have positively evaluated the effect of satavaptan in combination with conventional diuretics for the short-term treatment of ascites in patients with liver cirrhosis [95]. In patients with cirrhotic ascites, lixivaptan increased water excretion in a dose-dependent manner, reaching peak serum concentration one hour after oral administration [96]. However, a 2012 meta-analysis indicates that vaptans, including satavaptan and lixivaptan, do not affect mortality and offer only minor benefits in the treatment of ascites, which has discouraged further research in this area [8]. Furthermore, a multicenter study of patients with liver cirrhosis demonstrated that satavaptan has no impact on the incidence of hepatic encephalopathy [97].

### 4.5. Vaptans at the Extremes of Age: Pediatric and Geriatric Populations

Evidence regarding the use of vasopressin receptor antagonists in pediatric patients remains limited, mainly because ethical and methodological challenges hinder the conduct of adequately powered clinical trials in children. Consequently, current evidence is based largely on case reports, small retrospective studies, and only one randomized controlled trial. A 2022 systematic review including 226 pediatric patients concluded that vaptans appear to be safe and effective for the treatment of edema and hyponatremia; however, the quality of available evidence remains low, and no clear superiority over conventional therapy has been demonstrated [98]. In children with ADPKD, the first phase 3b randomized trial demonstrated that tolvaptan effectively suppressed vasopressin activity and showed a trend toward slowing kidney growth while maintaining a favorable safety profile without significant hepatotoxicity [99]. Beyond ADPKD, retrospective studies suggest that tolvaptan may improve diuresis and correct hyponatremia in children after congenital heart surgery, although these findings are based on relatively small cohorts [100]. More recently, a study of pediatric SIADH-associated hyponatremia demonstrated effective correction of serum sodium without cases of overly rapid correction; however, the authors emphasized the need for larger prospective studies before broader clinical implementation [79]. Overall, the available evidence suggests promising efficacy and safety of tolvaptan in pediatric patients, but larger multicenter prospective studies, particularly in neonates and children, are still required to establish its long-term efficacy, safety, and optimal place in clinical practice.

Evidence in elderly patients is also relatively limited and derives predominantly from retrospective studies in patients with acute decompensated heart failure. Available data suggest that early initiation of tolvaptan may shorten hospital stay and appears to be well tolerated even in very elderly patients, although predictors of treatment response and clinical outcomes remain incompletely understood [101,102,103]. Therefore, further prospective studies are needed to better define the efficacy and safety of tolvaptan in frail older adults.

## 5. Adverse Effects

**Figure 3:**
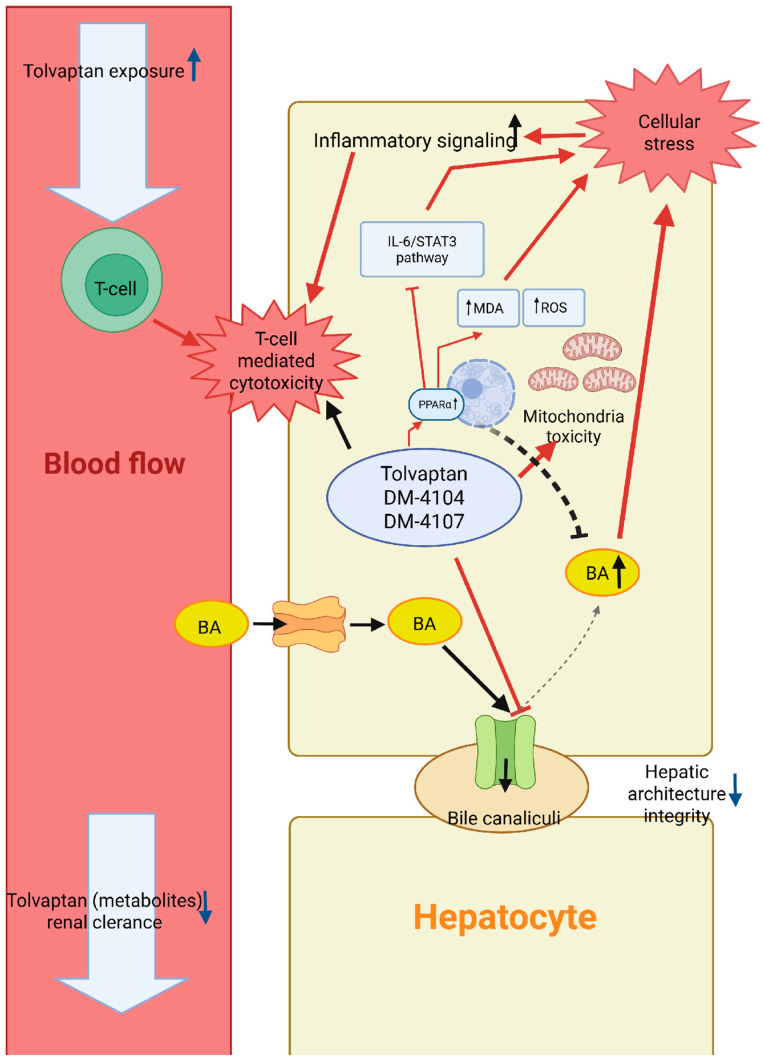
Examples of hypothetical pathways involved in hepatotoxicity. Tolvaptan and its metabolites reduce bile acid secretion [11], which, in the presence of an intact enterohepatic circulation, results in bile acid accumulation. Patients who develop drug-induced liver injury (DILI) exhibit increased PPARα expression [105]. Under physiological conditions, activation of PPARα would be expected to reduce bile acid synthesis while promoting their secretion [111]. However, activation of this transcription factor has also been associated with increased levels of malondialdehyde (MDA), a marker of lipid peroxidation, and dihydroethidium (DHE), reflecting enhanced reactive oxygen species (ROS) generation, as well as with suppression of the IL-6/STAT3 signaling pathway [112]. Collectively, these alterations may lead to increased cellular stress and activation of cytotoxic T cells. Created in BioRender. Sławiński, M. (2026) https://BioRender.com/3klva0n (accessed on 29 July 2026).

The use of tolvaptan is associated with several adverse effects. These are most frequently documented in patients with ADPKD, as this group requires long-term therapy. A primary concern is the elevated risk of idiosyncratic drug-induced liver injury (DILI). In accordance with Hy’s law criteria, drug-induced hepatocellular injury is defined by a triad: serum aminotransferase activity (ALT or AST) greater than 3× the upper limit of normal (ULN), concurrent elevation of total bilirubin to greater than 2× ULN, and the absence of initial cholestasis (indicated by alkaline phosphatase < 2× ULN) or alternative explanatory etiologies for acute liver injury [104]. Approximately 5% of patients treated with tolvaptan exhibit aminotransferase levels more than three times the ULN. In the TEMPO 3:4 and TEMPO 4:4 trials, 4.4% of subjects using the drug experienced an ALT increase > 3× ULN at least once, compared to 1% in the placebo group [45,47]. Transaminase elevations typically occur within the first 18 months of treatment [104]. In recent years, the mechanism of liver injury has been more precisely described. The metabolite DM-4103 inhibits hepatic proteins involved in bile acid transport, which may negatively affect bile acid homeostasis and impair their transport [23]. Furthermore, Yang et al. [105] identified 23 immune-related genes whose changes in expression are linked to the pathophysiology of DILI, with ICAM1, CXCL10, IGF1, CX3CL1, and EGFR being the most significant. In line with an immune-mediated mechanism, Hammond et al. [35] demonstrated that tolvaptan-induced hepatotoxicity involves the activation of drug-responsive T-cell clones, further underscoring the role of cellular immune responses in its pathophysiology. We presented a proposed hypothetical scheme of tolvaptan-induced liver injury in Figure 3. Consequently, strict patient monitoring guidelines have been implemented [106]. Monthly monitoring of liver parameters during the first 18 months is justified [107,108], as it significantly enhances both treatment safety and patient quality of life [109]. Isolated cases necessitating liver transplantation in ADPKD patients have been reported [110]. It is also worth noting that tolvaptan administration is frequently associated with increased water excretion, leading to thirst, dry mouth, polyuria, and nycturia. For some trial participants, these effects were overly bothersome. This led to a higher rate of treatment discontinuation in the tolvaptan group compared to the placebo group in the TEMPO 3:4 study [45].

The occurrence of adverse effects associated with conivaptan administration is primarily related to increased water excretion. As with tolvaptan, monitoring serum sodium levels is critical due to the risk of overly rapid correction [31,82]. During treatment, patients reported increased thirst and polyuria. In the case of intravenous administration, a significant issue was injection site reactions, manifesting as pain, edema, or phlebitis (inflammation of the vein) [81]. Furthermore, conivaptan acts as a potent inhibitor and a substrate of the CYP3A4 cytochrome P450 enzyme. It inhibits its own metabolism and increases the plasma concentrations of other drugs, which represents a significant limitation to its clinical use [32]. The most frequently reported drugs metabolized by this enzyme are listed in Table 4. The adverse effects of the other vaptans have been discussed in the preceding sections of this work.

## 6. Conclusions and Future Perspectives

Initially, the discovery of vaptans appeared to be the solution to many clinical challenges, offering hope for filling therapeutic gaps in conditions such as heart failure, liver cirrhosis, and SIADH. However, over time, they lost their perceived advantage in clinical trials due to insufficient clinical outcomes and high treatment costs compared to conventional medications [8,65,97]. Their use frequently remained limited to the clinical trial phase. Even when these drugs were approved for clinical use, their availability was restricted geographically: conivaptan is available in the United States, while mozavaptan is used in Japan [4,29].

Particular attention should be paid to tolvaptan, which is established in the treatment of ADPKD, hyponatremia secondary to SIADH, and volume overload-related heart failure. In Japan, it is also approved for the treatment of hepatic edema in the course of cirrhosis. Tolvaptan has filled a therapeutic gap for patients who do not respond adequately to conventional diuretics [69,70,71]. However, based on the experience of our center, we observe that some ADPKD patients do not respond to tolvaptan therapy. Research is ongoing to identify predictors of treatment response; however, available evidence remains strictly context-dependent and varies by clinical indication rather than reflecting universal determinants of vaptan response.

In cirrhotic ascites, negative or predictive physiological parameters include impaired renal function [115], the spot urine Na/K ratio [116], AQP2 concentration in urine [117], the stage of hepatocellular carcinoma [118], plasma renin activity [119] and baseline physical features such as higher body weight combined with lower blood urea nitrogen (BUN) levels [120]. In ADPKD, treatment progress appears to depend on underlying genetics, as Sekine et al. [121] indicated that patients without truncating *PKD1* or *PKD2* mutations showed less pronounced clinical benefit, although this observation is limited by a small sample size (*n* = 18). In heart failure, Hoshikawa et al. [122] investigated the diverse response to tolvaptan by examining the impact of *CYP3A5* and *ABCB1* (a drug efflux transporter) genotypes. They demonstrated that in CYP3A5*3/*3 homozygotes, characterized by a near total absence of the functional protein, tolvaptan plasma concentrations were significantly higher than in other genotypes, resulting in a stronger therapeutic effect, whereas the *ABCB1* genotype did not significantly influence metabolism. Additionally, a report by Kawaratani et al. [123] involving a genome-wide association study (GWAS) in cirrhotic patients identified the rs2991364 polymorphism of the *SVEP1* gene as a predictor of a positive response. Patients with the CC genotype exhibited an 86% response rate, while the presence of the T allele was associated with poorer clinical outcomes or total non-responsiveness. It is critical to emphasize that these proposed pharmacogenetic associations and physiological biomarkers are exploratory, strictly indication-specific, and derived from relatively small, single-center cohorts. Consequently, they currently lack robust external validation and cannot be generalized across all vaptan indications. This field requires further research, as the number of non-responders evaluated in existing studies remains relatively small. Nevertheless, searching for answers in pharmacogenetics appears highly promising; identifying validated, context-specific factors that influence treatment response could shorten the duration of ineffective therapy, optimize dosage strategies, and significantly reduce the risk of adverse effects.

## Figures and Tables

**Table 1:** Summary of pharmacological profiles and regulatory status of selected vaptans.

Feature	Tolvaptan	Conivaptan	Lixivaptan	Satavaptan	Mozavaptan
Receptor selectivity	Selective V2	Non-selective V1a/V2	Selective V2	Selective V2	Selective V2
Route of Administration	Oral (p.o.)	Intravenous (i.v.)	Oral (p.o.)	Oral (p.o.)	Oral (p.o.)
FDA Status (USA)	Approved (2009)	Approved (2005)	Not approved	Not approved	Not approved
EMA Status (EU)	Approved (2009 for SIADH; 2015 for ADPKD)	Not approved	Not approved	Not approved	Not approved
Primary Indication	Hyponatremia (SIADH), ADPKD	Hyponatremia (hospitalized)	Clinical trials	Clinical trials	Clinical trials
Elimination (t ½)	approx. 12 h	approx. 5–8 h	approx. 7–10 h	approx. 14–17 h	approx. 3–5 h

**Table 2:** Criteria for the initiation of tolvaptan treatment based on the KDIGO 2025 Clinical Practice Guideline for the Evaluation, Management, and Treatment of Autosomal Dominant Polycystic Kidney Disease (ADPKD) [44].

Initiation of Tolvaptan Treatment Should Be Offered to Adults with ADPKD Who Meet the Following Criteria:
eGFR ≥ 25 mL/min per 1.73 m^2^
and
Risk of rapid disease progression as indicated by either: Mayo class 1C to 1E
or
Historical rate of eGFR decline (≥3 mL/min per 1.73 m^2^ per year)

**Table 3:** Characteristics of studies on tolvaptan use in cardiac surgery and aortic diseases.

Study (Author and Year)	Study Design	Study Group	Objective	Conclusions
Ito et al. (2016) [66]	Retrospective cohort study	99 patients undergoing cardiovascular surgery with CPB (51 TLV vs. 48 control)	To evaluate the efficacy of tolvaptan on postoperative fluid management.	Tolvaptan facilitated early improvement of postoperative fluid overload and proved to be an effective component of diuretic therapy.
Bellos et al. (2019) [67]	Systematic review and meta-analysis	759 patients undergoing cardiac surgery (397 TLV vs. 398 standard therapy)	To assess the efficacy and safety of tolvaptan in fluid management after cardiac surgery.	Tolvaptan effectively reduces body weight without impairing renal function, serving as a promising alternative to standard loop diuretics.
Matsuyama et al. (2016) [68]	Retrospective observational study	50 patients undergoing open-heart surgery (25 TLV vs. 25 control)	To evaluate the effects of short-term tolvaptan administration on postoperative fluid retention.	Adding TLV to standard diuretics increases urine output without renal dysfunction; significantly shorter time to return to baseline body weight.
Wang et al. (2023) [69]	Retrospective cohort study	45 patients after Stanford type A acute aortic dissection surgery (21 TLV vs. 24 control)	To evaluate the clinical effects of tolvaptan in patients with acute aortic dissection.	TLV is safe and may significantly reduce the incidence of postoperative paroxysmal atrial fibrillation.
Nakamura et al. (2020) [70]	Prospective randomized controlled trial (RCT)	166 patients undergoing open-heart surgery (83 TLV vs. 83 control)	To evaluate the effect of TLV on postoperative paroxysmal atrial fibrillation (POAF) and renal function.	A 7.5 mg dose of TLV was effective in treating fluid retention, reducing the incidence of POAF and worsening renal function (WRF).
Wu et al. (2022) [71]	Experimental study (animal model)	ApoE −/− male mice (8–10 weeks old), *n* = 45 (groups: control, AngII, AngII + TLV)	To investigate the effect of TLV on the development and rupture of abdominal aortic aneurysm (AAA) and dissection.	TLV inhibited AAA development and dissection by reducing macrophage infiltration and smooth muscle cell apoptosis (via a cAMP-independent pathway).

**Table 4:** Clinically relevant inhibitors [113] and inducers [114] of cytochrome P450 CYP3A4.

Inhibitors	Inducers
Clarithromycin, erythromycin, cyclosporin, diltiazem, verapamil, itraconazole, ketoconazole, indinavir, ritonavir grapefruit juice	Carbamazepine, phenobarbital, phenytoin, rifampin, Hypericum perforatum (St. John’s wort)
Influence on tolvaptan	
Elevated plasma concentration of tolvaptan	Reduced plasma concentration of tolvaptan

## Data Availability

Data sharing not applicable. No new data were created or analyzed in this study.

## Abbreviations

The following abbreviations are used in this manuscript:

ADH	Antidiuretic hormone
cAMP	Cyclic adenosine monophosphate
AQP2	aquaporin-2
AVP	Arginine vasopressin
FDA	U. S. Food and Drug Administration
EMA	European Medicines Agency
SIADH	Syndrome of inappropriate antidiuretic hormone secretion
ADPKD	Autosomal Dominant Polycystic Kidney Disease
V2R	Vasopressin receptor type 2
CKD	Chronic kidney disease
RRT	Renal replacement therapy
P-gp	P-glycoprotein
ERK	Extracellular Signal-Regulated Kinase
TKV	Total kidney volume
eGFR	Estimated Glomerular Filtration Rate
KDIGO	Kidney Disease Improving Global Outcomes
TLV	Total liver volume
SAH	Subarachnoid hemorrhage
CRP	C-reactive protein
PLR	Platelet-to-lymphocyte ratio
SII	Systemic inflammatory index
HF	Heart failure
CPB	Cardiopulmonary bypass
AF	Atrial fibrillation
RAAS	Renin–angiotensin–aldosterone system
AAA	Abdominal aortic aneurysm
HASMCs	Human aortic smooth muscle cells
MMP	Matrix metalloproteinase
POAF	Post-operative atrial fibrillation
RCT	Randomized controlled trial
ODS	Osmotic demyelination syndrome
DILI	Drug-induced liver injury
ULN	Upper limit of normal
BUN	Blood urea nitrogen
GWAS	Genome-wide association studies
SSTR	Somatostatin receptor
SST	Somatostatin
AC	Adenylyl cyclase
ATP	Adenosine triphosphate
PC1	Policystyn 1
PC2	Policystyn 2
Il-6	Interleukin 6
STAT3	Signal transducer and activator of transcription 3
MDA	Malondialdehyde
PPARα	Peroxisome proliferator-activated receptor alpha
BA	Bile acids
PKA	Protein kinase A
ROS	Reactive oxygen species
